# Clinicians’ perspectives on a primary healthcare intervention to reduce antibiotic prescription for acute lower respiratory tract infections in Barcelona (Spain): a qualitative study

**DOI:** 10.1017/S1463423625000313

**Published:** 2025-07-04

**Authors:** Andrea García-Egea, Ana García-Sangenís, Carl Llor, Anna Berenguera, Ana Moragas, Ramon Monfà, Marta Trapero-Bertrán, Antoni Sisó-Almirall, Rosa Morros, Laura Medina-Perucha

**Affiliations:** 1 Fundació Institut Universitari per a la Recerca a l’Atenció Primària de Salut Jordi Gol i Gurina (IDIAPJGol), Barcelona, Spain; 2 Universitat Autònoma de Barcelona, Bellaterra (Cerdanyola del Vallès), Spain; 3 CIBER de Enfermedades Infecciosas, Instituto de Salud Carlos III, Madrid, Spain; 4 Department of Public Health, General Practice, University of Southern Denmark, Odense, Denmark; 5 Network for Research on Chronicity, Primary Care and Health Promotion (RICAPPS), Spain; 6 Universitat Rovira i Virgili, Reus, Spain; 7 Jaume I Health Centre, Institut Català de la Salut, Tarragona, Spain; 8 Plataforma SCReN, UICEC IDIAPJGol, Barcelona, Spain; 9 Basic Sciences Department, Universitat Internacional de Catalunya (UIC), Barcelona, Spain; 10 Consorci d’Atenció Primària de Salut Barcelona Esquerre (CAPSBE), Barcelona, Spain; 11 Catalan Society of Family and Community Medicine (CAMFiC), Catalonia, Spain

**Keywords:** antibiotics, communication skills, CRP testing, perceptions, prescribing, primary healthcare, qualitative research, respiratory tract infections

## Abstract

**Background:**

Interventions based on testing and communication training have been developed to reduce antibiotic prescribing in primary healthcare (PHC) for the treatment of acute lower respiratory infections (ALRTIs). However, research based on the experiences of PHC clinicians participating in ALTRIs interventions to reduce antibiotic prescribing in Barcelona is scanty.

**Aim:**

This study aimed to explore the perceptions and experiences of clinicians (physicians and nurses) on an intervention to reduce antibiotic prescription in PHC in Barcelona (Spain). This intervention was a randomised controlled study (cRCT) based on three arms: 1) use of a C-reactive protein (CRP) rapid test; 2) enhanced communication skills; and 3) combination of CRP rapid test and enhanced communication skills. In addition, the study aimed to explore the impact of COVID-19 on the detection of ALRTIs.

**Methods:**

This qualitative study used a socio-constructivist perspective. Sampling was purposive. Participants were selected based on age, sex, profession, intervention trial arm in which they participated, and the socioeconomic area of the PHC where they worked. They were recruited through the healthcare centres participating in the study. Nine participants (7 women and 2 men) participated in two focus groups, lasting 65–66 min, in September–October 2022. Framework analysis was used to analyse the data.

**Findings:**

Three themes were identified: *‘(The intervention) gave us reassurance’: intervention experiences among health professionals*. This theme includes accounts of clinicians’ satisfaction with the intervention, particularly with CRP testing to support clinical diagnoses; *‘We don’t have time in primary healthcare’: structural and community resources in healthcare services*. This theme encompasses clinicians’ experiences on healthcare pressures and PHC organisational structures barriers to PHC interventions; and *‘I only did three CRP’: impact of COVID-19 pandemic on the intervention*. The last theme focuses on the impact of the COVID-19 pandemic on the intervention’s implementation.

**Conclusions:**

CPR testing and promoting communication skills can be useful tools to support clinical decisions for ALRTIs. Structural barriers (e.g., healthcare pressures) and social inequities amongst service users were acknowledged as the main barriers for the implementation of ALRTIs interventions.

## Introduction

Antimicrobial resistance (AMR) constitutes one of the major public health threats of the 21st century. Globalisation, environmental adaptation of microbials, overuse, misuse, and clinical overprescription of antimicrobials are just some of the drivers contributing to the AMR crisis (Michael, Dominey-Howes and Labbate, 2014). Primary healthcare (PHC) is responsible for most of the antibiotics prescribed, mainly for the treatment of suspected acute lower respiratory infections (ALRTIs) (Costelloe *et al.*, 2010; Gulliford *et al.*, 2014; Meeker *et al.*, 2016). Indeed, 60% of ALRTIs in Catalonia, Spain, are treated with antibiotics constituting one of the areas with the highest rates in the European Union (Llor *et al.*, 2014).

Research in the United States, the United Kingdom, Germany, Australia, Ireland, Sweden, and Singapore has studied the experiences of clinicians in relation to antibiotic prescribing for ALRTIs. It has been described that ‘patient expectations’ are the main reason for antibiotic prescribing (Brookes-Howell *et al.*, 2012; Chan *et al.*, 2019; Hruza *et al.*, 2020; Patel *et al.*, 2020). Another reason reported in the literature is that clinicians want to avoid the degradation of relationships with patients and unsatisfaction with clinical visits (Patel *et al.*, 2020). Other reasons include diagnostic (Brookes-Howell *et al.*, 2012; Fletcher-Lartey *et al.*, 2016; Chan *et al.*, 2019; Hruza *et al.*, 2020; Patel *et al.*, 2020) and prognostic uncertainty (Hosoglu, Classen and Akturk, 2021), time constraints due to workload (Fletcher-Lartey *et al.*, 2016; Patel *et al.*, 2020), lack of testing facilities and standardised guidelines (Brookes-Howell *et al.*, 2012; O’Doherty *et al.*, 2019; Hruza *et al.*, 2020), and poor organisation in healthcare centres (e.g., lack of nurse triage, lack of leadership in the job environment) (Dempsey *et al.*, 2014; Strandberg *et al.*, 2016).

Educational interventions, procalcitonin testing (Schuetz *et al.*, 2012, 2018), communication training among clinicians (Van der Velden *et al.*, 2012), delayed prescribing or C-reactive protein (CRP) measurement (Llor *et al.*, 2012; Minnaard *et al.*, 2016; Smedemark *et al.*, 2022; Zhang *et al.*, 2022) are evidence-based interventions aimed at reducing antibiotic prescribing in ALRTIs. CRP testing may facilitate timely and well-informed clinicians’ choices regarding antibiotic prescriptions (Boere *et al.*, 2021). Moreover, research has revealed that CRP enhances a more accurate diagnostic in the prediction of bacterial ALRTIs in contrast to procalcitonin testing (Van Vugt *et al.*, 2013). Multifaceted interventions (e.g., clinician communication training combined with CRP testing) can achieve important reductions in antibiotic prescribing for respiratory tract infections (Little *et al.*, 2013). Evidence shows that CRP testing and clinician communication skills training are cost-effective interventions and both combined reduce antibiotic prescribing maintaining patients’ satisfaction with healthcare (Cals *et al.*, 2011).

Although some evidence has highlighted clinicians’ experiences related to antibiotic prescription in ALRTIs, little is known about the perceptions of clinicians following participation in an intervention programme focused on reducing antibiotic prescription in PHC in Barcelona (Spain). For this reason, the present study aimed to explore the perceptions and experiences of PHC clinicians (physicians and nurses) on an intervention to reduce antibiotic prescription in PHC in Barcelona (Spain). This intervention was a randomised controlled study (cRCT) based on three arms to reduce antibiotic prescription in ALRTIs: 1) use of a CRP rapid test; 2) enhanced communication skills; and 3) combination of CRP rapid test and enhanced communication skills. In addition, the study aimed to explore the impact of COVID-19 on the detection of ALRTIs. Concretely, this study also aimed to explore clinicians’ perceptions on the barriers and facilitators to implement interventions to reduce antibiotic prescription in PHC.

This qualitative study is part of the ISAAC-CAT project, an embedded mixed-method study including a cluster randomised clinical trial (cRCT) that aimed to evaluate the effectiveness and efficiency of three interventions to reduce antibiotic prescribing in patients with ALRTIs in Catalan PHC (Ruiz *et al.*, 2019). Before the intervention, clinicians (both physicians and nurses) of three intervention arms received face-to-face two-hour training workshop. This training was followed by monthly internet training on how to target testing (e.g., in cases of clinical uncertainty) and how to negotiate clinical decisions with service users. Both nurses and physicians were equally engaged in the intervention, with the sole distinction that physicians were able to prescribe antibiotics.

A pre-intervention qualitative investigation was undertaken to provide insights for the development of the cRCT (Medina-Perucha *et al.*, 2020). A post-intervention qualitative study (presented in this article) was conducted to comprehend the clinicians’ perceptions, as well as the facilitators and barriers, that face with the incorporation of these interventions in their routinary practices.

## Methods

### Study design

This qualitative study used a socio-constructivist framework, considering that experiences and attitudes are generated and modulated through social processes (Berenguera Ossó *et al.*, 2017).

### Participants

The study participants (*N* = 9) were five physicians and four nurses who had taken part in the ISAAC-CAT cRCT study and who came from five PHC centres from Barcelona (Spain). Three physicians and nurses had participated in CRP testing intervention arm, one participant in the enhanced communication skills arm, and five participants in the third intervention arm, which included CPR testing and communication skills. Further details on the characteristics of the participants are available in Table 1.

**Table 1.** Participants’ sociodemographic characteristics (*N* = 9)

ID	Age	Gender	Profession	Localisation of PHC based on basic health areas^*^	Intervention arm	Focus group in which they participated
P1	54	Woman	Physician	Barcelona 1-B	CRP + communication skills	First
P2	33	Man	Physician	Barcelona 1-B	CRP + communication skills	First
P3	49	Woman	Nurse	Barcelona 7-A	CRP + communication skills	First
P4	51	Woman	Physician	Barcelona 7-E	CRP	First
P5	45	Woman	Nurse	Barcelona 7-E	CRP	First
P6	29	Woman	Nurse	Barcelona 7-A	CRP + communication skills	Second
P7	47	Woman	Physician	Barcelona 7-A	CRP + communication skills	Second
P8	43	Woman	Nurse	Barcelona 9-H	Communication skills	Second
P9	48	Man	Physician	Barcelona 5-A and 5-B	CRP	Second

* Barcelona is divided into basic health areas. The localisation of PHC where professionals work is specified according to the basic health areas in the city.

### Sampling, recruitment and data collection

Sampling was selective and purposive. We followed quota sampling as a method to ensure the inclusion of participants that represented the entire group participating in the intervention (Luborsky and Rubinstein, 1995). Participants were selected based on age, sex, profession, intervention trial arm in which they participated, and the socioeconomic area of the PHC where they worked. Seventy-seven clinicians from twenty PHC of Barcelona participating in the cRCT of the ISAAC-CAT study were invited to participate in the qualitative study. Participants were contacted via email by one of the researchers (AGS), to invite them to take part into the post-intervention qualitative study. Despite the efforts made by the research team, only nine people take part in the study. Reasons for refusal to participate were mostly related to time constraints and workload pressures. Also, clinicians’ commitment to the study was impaired given that the study could not be finalised due to the COVID-19 pandemic. Four participants did not attend focus groups after accepting to participate due to last-minute schedule conflicts. While the team tried to enrol more participants to conduct more focus groups this was not possible due to the reluctance for clinicians to participate (for the reasons already stated) and time limitations with funders.

Two focus groups were conducted between September and October 2022. The focus group technique was chosen to explore individual discourses in groups and to stimulate the emergence of experiences due to participant interactions. Also because it explores individual discourses in groups regarding a particular topic (Glitz, 1997). A semi-structured topic guide was developed by the research team, based on their experience on qualitative research and following the study objectives (see Additional file 1). The focus groups were conducted face-to-face on two different days at the research centre. P1 to P5 were involved in the first focus group, whereas P6 to P9 took part in the second focus group. Data saturation was reached, as the same themes in discourses were repeated between participants in the two focus groups.

The researcher LMP conducted the groups and AGE acted as an observer, writing field notes, and sometimes intervening. LMP (PhD) have over eight years of experience conducting qualitative and mixed-methods. AGE (MPH) has worked as a research assistant in qualitative and mixed-methods research for over a year. The research team is committed to conducting public health research from a social determinant of health and gender-based perspective and is focused on PHC and AMR research. The study participants were informed about the interviewers’ work field and their motivation for doing this research. It is important to mention that there was no prior relationship between the investigators who conducted the data collection (AGE and LMP) and the participants. All participants provided verbal and written consent and completed a brief questionnaire on sociodemographic characteristics (see Table 1). Both focus groups lasted 65 and 66 min and were audio-recorded and transcribed verbatim. Transcripts were not returned to participants and participants did not provide feedback on the findings. A 40€ voucher was provided to all participants as a token of thanks.

### Data analysis

Data were analysed using Framework Analysis, an approach that structures data based on a hierarchical thematic framework (Ritchie and Spencer, 1994). The steps of Framework Analysis are the following: 1) familiarisation with the data through re-listening of the audio-recordings and re-reading the transcripts; 2) developing a working analytical framework based on the first step, previous evidence and the aims of the study; 3) applying the analytical framework; for instance, indexing transcripts to the framework developed; 4) charting data into the framework matrix, which involves summarising the data from each transcript in categories, and 5) interpreting the data through discussions with other members of the research team and mapping connections between different categories. The framework was constructed based on the themes: 1) ‘(The intervention) gave us reassurance’: intervention experiences among health professionals; 2) ‘We don’t have time in primary healthcare’: structural and community resources in healthcare services; and 3) ‘I only did three CRP’: impact of COVID-19 pandemic on the intervention. Then, subthemes were inductively identified. Data analysis was performed by AGE and LMP. Then, a meeting with the research team was carried out to present the themes and subthemes found by the analysis, in order to discuss the results and how to present them. The research team reached the consensus during the meeting.

## Results

Three main themes were identified: “‘(The intervention) gave us reassurance’: intervention experiences among health professionals”, “‘We don’t have time in primary healthcare’: structural and community resources in healthcare services” and “‘I only did three CRP’: impact of COVID-19 pandemic on the intervention”. There were, respectively, two, three and two subthemes for the first, second, and third themes.

### ‘(The intervention) gave me reassurance’: intervention experiences among health professionals

#### CRP as a tool to reinforce clinical decisions

Participants expressed overall satisfaction with the three interventions. Concretely, participants reported contentment with CRP testing. They mentioned an increased sense of security, reinforcement of their clinical decisions, or the CRP test result even often acted as a deciding factor in their final diagnosis:

*‘This happened to me with a patient with bronchiectasis (…), as you might suspect that he has pneumonia. (…) I did a CRP and the result was a low level. (…) I remember this because it helped me a lot. If it had been for the auscultation, I would have put an antibiotic or an X-ray directly’ - P2, 33 years, men, physician.*

Another positive impact of CRP was the improvement in the relationship between clinicians and patients. Participants felt that this change was especially notable among patients who were more insistent about the demand for antibiotics, with these patients being more easily convinced of antibiotic prescription based on a diagnostic test. Also, they considered CRP as a cheaper, faster, more accessible, and less invasive test in comparison to other ALRTI testing methods (e.g., X-Ray):

*‘Maybe you don’t have to go to other levels of testing, for example, an X-ray. (…) you can do a CRP, which would be a much lower level and more economical, (…), it’s also much quicker’ - P5, 45 years, woman, nurse.*

By contrast, clinicians complained about the occurrence of technical errors (e.g., wrong temperature of the reagents in the diagnostic machine). However, these were solved quickly and without technical assistance:

*‘…but basically, what failed us a lot was the machine. The machine and the reagents were a barrier for us’ - P3, 49 years, woman, nurse.*

#### Communication skills as a support to CRP results

Professionals participating in the arm which combined both CRP and communication skills, stated that communication skills generally supported the results provided by CRP. For them the enhancement of communication skills was interesting but did not have the same persuasive power as the CRP, especially in patients who insisted on antibiotic prescription.

Participants mentioned that communication skills are already intrinsic to clinicians, due to their professional role. In contrast, others felt that good communication abilities are not strictly related to their role. In line with this, training the healthcare professionals in communication skills during their professional career was important, as these abilities could be implemented after the intervention:

*‘I was hoping to do the communication skills training, because in the end this technique is very important when communicating that you don’t give an antibiotic to a person who comes to you to get an antibiotic, but I would like to have communicative skill, because a lot of times…We don’t have CRP right now, and we continue seeing bronchitis’ - P9, 48 years, man, physician.*

### ‘We don’t have time in primary healthcare’: structural and community resources in healthcare services

#### Healthcare pressure as a barrier for intervention implementation

Participants stated that the intervention was often complicated to carry out due to the PHC pressure. They suggested that clinicians could invest more time implementing interventions such as these if they were not assigned bureaucratic tasks:

*‘If we didn’t have all the pressure of sick leave and all these people who come to ask for sick leave for a day or two because they are ill, you could stop and explain to the patient that (…). And with these people, (…) you can stop calmly to explain “look, you don’t need antibiotics”. But, of course, the pressure of assistance…’-P2, 33 years, man, physician.*

The implementation of certain interventions was perceived as a feeling of even more pressure and extra work. For this reason, they proposed interventions adapted to the time and human sources available in PHC.

#### The impact of healthcare centre structures on the intervention

Participants explained that PHC was organised differently (e.g., existence or not of emergency department). In line with this, they commented on the difficulties encountered in recruiting patients to participate in the study in emergency departments. By contrast, the recruitment of patients in their own consultations was perceived to be easier. In these situations, the patient already knew the clinician, and therefore there was already a relationship based on trust:

*‘Well, I think that patients from the clinician’s own list probably have more trust (…). If it’s a patient you’ve found in the emergency room and you have to perform the intervention at that moment, you have to ask the professional… In any case, with their doctor’. - P1, 54 years, woman, physician.*

The lack of involvement of all clinicians (and other PHC staff) represented a barrier for implementation since only a few professionals involved in the intervention were able to apply CPR or communication skills in patients. For this reason, participating clinicians were called by non-participating clinicians to, for example, perform a CRP when an ALTRI was suspected.

#### Social inequities as relevant to implementing primary healthcare interventions

Differences in population cultural resources – depending on their geographical location in the city – were acknowledged by two participants. For example, access to health literacy was very different depending on the population’s educational resources. Based on participants’ accounts, the use of an antibiotic was better understood among patients who lived in higher socioeconomic areas. In addition, based on their experience, these patients were less demanding of antibiotic prescriptions:

*‘[PHC centre], which is located in [high socioeconomic neighbourhood] (…), is one of the easiest places for the population to have cultural resources; they have a greater capacity for understanding, and although it may have its prejudices and its experiences, it is more capable of understanding a refusal of antibiotics, which, compared to where I worked before in [low socioeconomic neighbourhood], it was much more difficult. (…) They don’t differentiate a virus from a bacterium’ - P9, 48 years, man,physician.*

Participants commented that those with fewer economic resources perceived clinicians as an essential (and unique) figure to address their health. In contrast, those with more economic resources saw clinicians from the public health service as a non-essential figure, as they could access private healthcare:

*‘For some people we (clinicians) are the beginning and the end; for some people we are something else on their scale: I start here but if you don’t give it (the antibiotic) to me, I’ll go somewhere else. And of course, there are people who are “you give it to me or …. (I have no choice)”’- P8, 43 years, woman, nurse.*

### ‘I only did three CRP’: the impact of the COVID-19 pandemic on the intervention

#### Research disrupted due to the COVID-19 pandemic

Participants’ comments were strongly marked by their experiences during the COVID-19 pandemic. The recruiting period started in November 2019 should have run for 16 months. However, in February 2020, it was terminated early due to the outbreak of the COVID-19 pandemic. In fact, the ISAAC-CAT study had to be stopped during this period. They added that the intervention would have gone more smoothly (e.g., easier to implement the CRP testing) if its implementation course had continued.

Participants mentioned some lessons learned from the COVID-19 pandemic. For instance, the pandemic had somehow helped to reduce patient demands for antibiotic prescriptions, since many people understood the differences between viruses and bacteria:

*‘And I also think that COVID-19, (…), but it has changed the dynamics a little. The patient has understood very well what a virus is and it can’t be treated with anything. (…)’ - P2, 33 years, man, physician.*

#### The COVID-19 pandemic as a barrier for primary healthcare research

Participants mentioned a general lack of implication of clinicians in research in general but also during the COVID-19 pandemic, especially professionals working in PHC. Moreover, the development of research projects during this period was even more reduced, except for investigations directly related to COVID-19.

PHC was overwhelmed due to the pandemic. Clinicians’ tasks were also reformulated during the pandemic and were basically focused on treating patients with COVID-19 and other bureaucratic issues, such as requesting sick leaves. Thus, they complained that they had no time to develop and propose investigations:

*‘If you have the ability to observe from the outside, you can clearly see what is happening (…), you can ask yourself questions, we can think. If you go on doing administrative tasks, that don’t fulfil you, you miss things’-P7, 47 years, women, physician.*

The COVID-19 pandemic was seen as a learning opportunity for future pandemics, suggesting that research could strengthen knowledge for better internal organisation and restructuration of tasks in PHC:

*‘I think if there was another pandemic, we would obviously know better how to manage it and I think this is where we could exploit research a little, that it would serve for something. From all the bad, you can certainly get information and things that we could consider if it happened again’. -P3, 49 years, woman, nurse.*

## Discussion

In our study, clinicians expressed their satisfaction, independently of the intervention arm in which they participated. These findings are consistent with previous studies, which have also shown that these interventions are positively valued by clinicians (Hardy *et al.*, 2017; Eley *et al.*, 2018; Phillips *et al.*, 2020). Several studies have reported that the use of multifaceted interventions, such as CRP testing and training in enhanced communication skills can significantly reduce antibiotic prescribing (Cals *et al.*, 2009; Llor *et al.*, 2012).

Based on the clinicians’ accounts, CRP testing seemed to improve appropriate antibiotic prescribing due to its supportive role in the diagnostic process and empowering clinicians confidence (Cals *et al.*, 2009; Hardy *et al.*, 2017; Eley *et al.*, 2018; Phillips *et al.*, 2020). As the study participants mentioned, CRP testing can prevent the use of other diagnostics, such as imaging studies (e.g., chest X-ray). Although chest X-ray is commonly used as a supportive diagnostic tool, it is sometimes far from being used due to low availability, high radiation, and high costs for healthcare systems (Hansen *et al.*, 2020). Incorporating the use of less invasive and more cost-effective diagnostic tools in PHC is therefore imperative.

The perceptions and experiences of the clinicians in relation to communication skills interventions could not be explored in depth. However, most participants felt that communication skills were important in their clinical practice to communicate and negotiate, for instance, the adequacy of antibiotic prescriptions with patients. In fact, training in communication skills is a promising strategy to promote awareness of adequate antibiotic use (Cals *et al.*, 2009; Llor *et al.*, 2012; Hruza *et al.*, 2020). This requires horizontal and clear communication by, for instance, adapting medical terminology to more colloquial languages (Saukko *et al.*, 2019) or the provision of more succinct messages (Van der Velden *et al.*, 2012). Also, the active contribution of patients to their own health decisions promotes patient empowerment (Medina-Perucha *et al.*, 2020).

Socioeconomic diversity across service users needs to be considered, as patients with lower education and those living in more deprived socioeconomic contexts are more likely to be prescribed antibiotics for ALRTIs (Kumar, Little and Britten, 2003; Saukko *et al.*, 2019). Moreover, individuals in more deprived communities can only often access public healthcare systems and hold belief systems that may make them more insistent for antibiotic prescription. On the other hand, having the socioeconomic resources to access private healthcare may make more privileged communities be less insistent on antibiotic prescriptions in public healthcare consultations. This can be explained as on-demand prescriptions are more common in private practices. In fact, studies conducted in Ireland, China, and Malaysia have found that general practitioners felt more pressured to meet the expectations of fee-paying patients (Ab Rahman, Teng and Sivasampu, 2016; O’Doherty *et al.*, 2019). This point reinforces the need to foster communication skills among clinicians. It also suggests the need to consider how the increased privatisation of healthcare systems (including in Catalonia) can significantly and negatively impact health equity through differential access to healthcare systems based on socioeconomic factors (Schenkman and Moraes Bousquat, 2019). Indeed, some factors influencing the population’s use of and resistance to antibiotics are income, impaired access to healthcare, gender, religion, culture, educational status, and disparities in health literacy based on socioeconomic factors (Masiero *et al.*, 2010; Charani *et al.*, 2021). In order to adapt and develop interventions that consider different populations, future interventions should consider social determinants of health.

Moreover, the systemic lack of resources for public PHC should be discussed. Participants mentioned the importance of considering the impact of work pressure and burnout (Hosoglu, Classen and Akturk, 2021) related to time constraints which may limit the quality of their clinical care (Schuetz *et al.*, 2018). Supported by other evidence, this can greatly modulate the development and implementation of interventions in PHC, especially those that require more training and time to implement (e.g., communication skills).

The ISAAC-CAT intervention was implemented for a short time due to the COVID-19 pandemic outbreak. Clinicians involved in the present study recognise the difficulties to prioritise research over clinical pressures during the pandemic (Czeisler *et al.*, 2020). In line with this, and as supported by the narratives of the participants, a well-functioning PHC system needs to be ensured to provide high-quality clinical practice and incorporate research activities in PHC (Hanson *et al.*, 2022). For this reason, policymakers should also address systemic barriers for the adequate functioning of PHC.

To our knowledge, this is the first qualitative data on the perceptions of clinicians in an intervention based on CRP testing and enhanced communication for ALRTIs within the Spanish context. Another strength of this study is that the use of a qualitative methodology can provide insights on perceptions and experiences that quantitative research cannot offer. Conversely, some limitations need to be acknowledged. First, the limited acceptance to participate led to the misrepresentation of some profiles (e.g., male participants and participants of the communication skills intervention arm). Reasons for participation refusal were mostly related to time constraints and workload pressures. Another important barrier to sampling and recruitment was the timeframe the study funder allowed to complete the study. The ISAAC-CAT project was planned to be conducted between 2019 and 2022. This was greatly affected by funding and time pressures together with the impossibility to conduct the ISAAC-CAT study as planned due to the COVID-19 pandemic. Also, data saturation was compromised due to the short timeframe for the study, which greatly limited the extension and depth of the clinicians’ experiences. Lastly, the amount of the incentive might have encouraged participation for economic reasons. Despite the limitations described above, the results should be considered as a first approach of clinicians’ perceptions and experiences implementing PHC interventions for ALRTIs in Catalonia.

## Conclusion

Our findings suggest that PHC clinicians in Barcelona are satisfied with CRP testing and enhanced communication skills to aid clinical decisions to treat ALRTIs in PHC. Both interventions were useful to reassure professionals regarding their clinical decisions and promote clinician–patient communication and shared decision-making. However, structural barriers (e.g., healthcare pressure, internal organisation of PHC) and social inequities of health are challenging factors when implementing interventions. The impact of COVID-19 (and other potential future pandemics) on research and medical practice needs to be considered. Future research should focus on assessing CRP testing and communication skills in rural areas and consider social inequities of health, by including vulnerable and hard-to-reach populations. The perspectives of patients should also be included in future studies. Overall, the results of this research support the use of CRP testing and communication skills as routine tools to ensure adequate antibiotic prescribing for ALRTIs in PHC in Catalonia.

## Supplementary material

The supplementary material for this article can be found at https://doi.org/10.1017/S1463423625000313

## Supporting information

García-Egea et al. supplementary material García-Egea et al. supplementary material
