# Supplementary material for: Clinicians’ perspectives on a primary healthcare intervention to reduce antibiotic prescription for acute lower respiratory tract infections in Barcelona (Spain): a qualitative study
Source: Prim Health Care Res Dev. 2025 Jul 4;26:e54. doi: 10.1017/S1463423625000313 (PMC12260738; doi:10.1017/S1463423625000313)
Supplement: García-Egea et al. supplementary material [file S1463423625000313sup001.docx]

**Additional file 1.** Interview topic guide.

1. Reception and greetings.
2. Introduce researchers and the institution.
3. Explain the objectives of the study and the focus group.
4. Provide the information sheet, give time to read it and ask questions (if they want to).
5. Provide the sociodemographic questionnaire.
6. Remind about confidentiality of the focus group. Also, the anonymity during analysis and dissemination phases. Request signed informed consent.
7. Ask for consent to audio-record the interview.
8. Presentation of each participant of the focus group (e.g. name, occupation, centre where they work and the intervention trial arm they applied)
9. Study questions regarding to the objectives:

| **QUESTIONS** |
| --- |
| 1. In general, what do you think about the interventions carried out by the ISAAC project?  - What aspects did you like? - What aspects of the intervention in which you participated do you think should be changed?  1. Patients were given leaflets during the consultation in two intervention trial arms. What do you think of these materials?  - What aspects did you like? - What would you have changed or added? - Do you think they have been effective?  1. What has been your experience applying the different intervention arms in your usual clinical practice?  - What drawbacks/difficulties have you seen when incorporating these interventions in clinical practice?  1. Several research indicate changes in relationships between clinicians and patients when new techniques are implemented in clinical practice. Could you explain to us if you have had any similar experience derived from this intervention? 2. What do you think about the sustainability of applying these interventions in the usual clinical practice? 3. We know that the pandemic had an impact on the detection of several pathologies. For this reason, we want to ask you: how do you think the COVID-19 pandemic has affected the detection of ALRTIs?  - How do you think that changes (e.g. organizational) in healthcare centres could affect the development of ISAAC project? - What are the barriers that you have encountered during this period to apply the different intervention trial arms?  1. Apart from the ISAAC project, what do you think about conducting primary healthcare research during a pandemic (in this case, the COVID-19 pandemic)?  - What difficulties have you encountered? - What facilitators have you encountered for developing research during the pandemic? - What changes might be necessary to improve primary healthcare research during a pandemic? |

10. Participants’ free contribution

- Would you like to add something else?

- Would you like to go deeper into any issue?

- Would you like to ask us any questions?

11. Thank them for participating

12. Give them the compensatory voucher
